# Clinically probable alkaptonuria-associated ochronosis presenting as multilevel cervical disc herniation with myeloradiculopathy managed by three-level C4–7 anterior cervical discectomy and fusion: a case report

**DOI:** 10.3389/fendo.2026.1805510

**Published:** 2026-06-15

**Authors:** Zichuan Wu, Baifeng Sun, Junzhe Sheng, Yong Hu, Yang Liu

**Affiliations:** 1 Department of Orthopedics, Ningbo No.6 Hospital, Ningbo, Zhejiang, China; 2 Ningbo Clinical Research Center for Orthopedics, Sports Medicine & Rehabilitation, Ningbo, Zhejiang, China; 3 Department of Spine Surgery, Changzheng Hospital Affiliated to the Naval Medical University, Shanghai, China

**Keywords:** alkaptonuria, anterior cervical discectomy and fusion, black disc, cervical disc herniation, cervical myelopathy, ochronosis

## Abstract

**Background/introduction:**

Alkaptonuria (AKU) is a rare disorder of tyrosine metabolism characterized by homogentisic acid accumulation and ochronotic pigment deposition in connective tissues. Spinal involvement is common, but cervical disc herniation with clinically significant myeloradiculopathy requiring multilevel anterior surgery is infrequently reported. A distinctive operative hallmark is the diffusely dark (“black”) intervertebral disc/nucleus pulposus, which may be the first clue to the underlying diagnosis.

**Case presentation:**

An adult patient presented with 6 months of progressive neck pain and bilateral upper-limb numbness/pain, most prominent in the lateral aspect of the right upper arm and involving all fingers of both hands, accompanied by gait disturbance described as a “cotton-like sensation.” Neurologic examination demonstrated long-tract signs consistent with cervical myelopathy and superimposed radiculopathy. Cervical MRI showed multilevel disc degeneration with posterior protrusions, ventral dural sac compression, and intramedullary signal change, with the most severe stenosis at C4/5, C5/6, and C6/7.

**Interventions:**

Given progressive myeloradiculopathy with multilevel anterior compression, the patient underwent three-level C4–7 anterior cervical discectomy and fusion (ACDF) to achieve ventral decompression and segmental stabilization. Intraoperatively, the intervertebral discs and nucleus pulposus were diffusely black-pigmented, a gross appearance that strongly raised suspicion for ochronotic disc involvement. Representative disc material was collected for microbiological testing, which showed no bacterial or fungal growth. Because quantitative urinary homogentisic acid measurement and HGD genetic testing were not completed before manuscript preparation, the diagnosis was regarded as clinically probable rather than biochemically confirmed.

**Results/conclusions:**

Postoperatively, the patient showed early improvement in limb numbness and gait stability, and radiographs confirmed satisfactory implant position and cervical alignment. This case emphasizes that a “black disc” encountered during routine cervical discectomy should prompt immediate consideration of AKU/ochronosis, careful documentation and tissue sampling, and definitive metabolic testing (quantitative urinary homogentisic acid measurement, with HGD genetic testing when available). However, in the absence of biochemical or genetic confirmation, the intraoperative black-disc finding should be interpreted as a highly suggestive diagnostic clue rather than definitive proof of AKU. Standard cervical decompression and fusion principles remain effective for neurologic compromise, but recognition of a possible underlying metabolic disorder is essential for systemic evaluation and long-term surveillance.

## Introduction

Alkaptonuria (AKU) is an ultra-rare autosomal recessive disorder of tyrosine metabolism caused by deficiency of homogentisate 1,2-dioxygenase, resulting in lifelong accumulation of homogentisic acid (HGA). Over decades, HGA undergoes oxidation and polymerization, forming ochronotic pigment that deposits preferentially in collagen-rich tissues (1). Clinically, this process underlies the classic triad of urine darkening, connective-tissue pigmentation, and a progressive, debilitating osteoarthropathy.

The spine is among the earliest and most consistently involved musculoskeletal sites in AKU (2). Ochronotic involvement of fibrocartilaginous structures promotes premature disc desiccation, fissuring, and mechanical failure, often accompanied by striking intervertebral disc calcification; with progression, disc collapse and segmental autofusion may occur. Radiographically, flattened and densely calcified discs—together with osteophyte formation and ligamentous calcification—are frequently emphasized as highly characteristic features of the disease and can be mistaken for more common degenerative spondylosis or even spondyloarthropathy when the systemic context is not recognized.

Although spinal pain and stiffness are well described in AKU, symptomatic disc herniation producing neurological compromise appears uncommon in the literature, particularly in the cervical spine. When present, the diagnosis may be first suspected intraoperatively, where surgeons encounter a conspicuously dark (“black”) intervertebral disc and nucleus pulposus—an appearance increasingly reported as a practical diagnostic clue in otherwise routine cervical discectomy cases (3–7).

Here, we report a patient with clinical features highly suggestive of systemic ochronosis who developed progressive cervical myeloradiculopathy due to multilevel cervical disc herniation and spinal canal compromise, ultimately requiring three-level C4–7 anterior cervical discectomy and fusion. Intraoperatively, the excised disc material and nucleus pulposus demonstrated marked black discoloration, providing an important visual clue to a probable underlying metabolic disorder.

## Case presentation

A 56-year-old woman with a 10-year history of hypertension (treated with amlodipine) presented with progressive neck pain and bilateral upper-limb numbness/pain for 6 months. The sensory symptoms were most prominent along the lateral aspect of the right upper arm and involved all fingers of both hands, occurring in persistent episodes. Over the preceding 2 months, she noted clear deterioration, accompanied by a gait disturbance described as a “cotton-like sensation” when walking, raising concern for evolving cervical myelopathy. She denied dizziness, headache, nausea, vomiting, chest tightness, or a truncal “band-like” sensation. She reported no history of trauma, smoking, or alcohol use, and her family history was noncontributory.

### Clinical findings

On admission, vital signs were stable (temperature 36.6 °C, blood pressure 130/80 mmHg). She ambulated into the ward with an unsteady gait. Cervical range of motion was not markedly restricted. Spurling test was positive bilaterally, consistent with concomitant radicular irritation. On sensory examination, decreased pinprick/light-touch and temperature sensation was noted over the pulp of all five fingertips bilaterally, while sensation over the trunk, perineum, and remaining extremities was largely preserved. Proprioception and vibration sense were not clearly impaired.

Motor testing demonstrated Medical Research Council (MRC) grade 5/5 strength in the deltoids, biceps, triceps, wrist flexors/extensors, and intrinsic hand muscles bilaterally. Lower-extremity strength was approximately 4/5 diffusely (including iliopsoas, quadriceps, hamstrings, tibialis anterior, gastrocnemius–soleus, and toe dorsiflexion/plantarflexion). Notably, she had fine motor impairment of both hands. Deep tendon reflexes were brisk in the upper and lower extremities; Hoffmann sign and Babinski sign were positive bilaterally, while ankle clonus was absent. These findings supported cervical spondylotic myeloradiculopathy.

### Diagnostic assessment

Preoperative sagittal T2-weighted cervical MRI demonstrated multilevel disc desiccation and posterior disc protrusions at C4/5, C5/6, and C6/7, causing ventral compression of the thecal sac and spinal cord. Short-segment intramedullary T2 hyperintensity was observed at the maximally compressed segment, supporting compressive cervical myelopathy (**Figure 1**). Sagittal cervical CT showed multilevel disc-space narrowing and endplate osteophyte formation across C4–7, further supporting chronic multilevel degenerative spondyloarthropathy with ventral canal compromise; in the context of the subsequent intraoperative and systemic findings, ochronotic involvement was clinically suspected (**Figure 2**).

**Figure 1:**
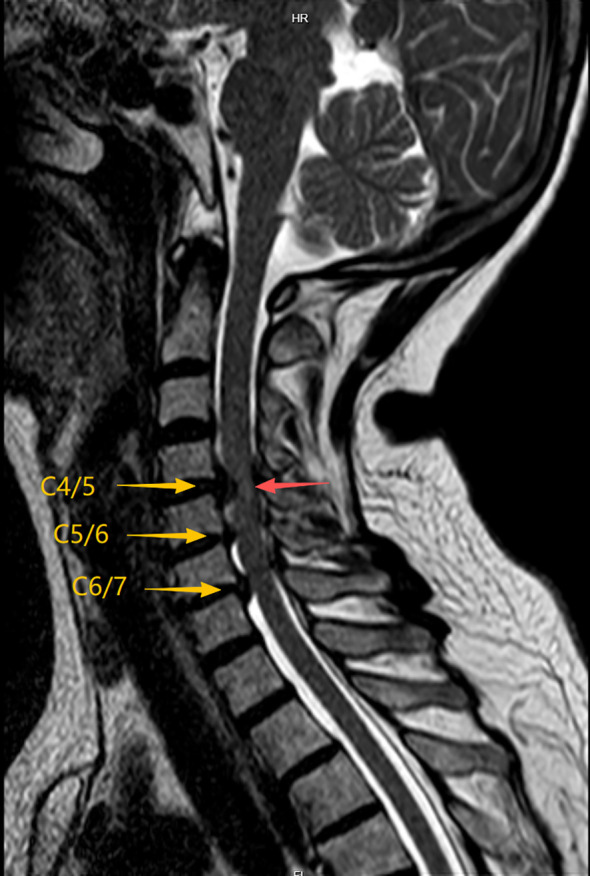
Preoperative sagittal T2-weighted cervical magnetic resonance imaging. Sagittal T2-weighted MRI demonstrates multilevel cervical disc degeneration with posterior disc protrusions at C4/5, C5/6, and C6/7. Yellow arrows indicate the principal ventral compression levels, and the red arrow indicates focal intramedullary T2 hyperintensity at the maximally compressed segment, consistent with compressive cervical myelopathy.

**Figure 2:**
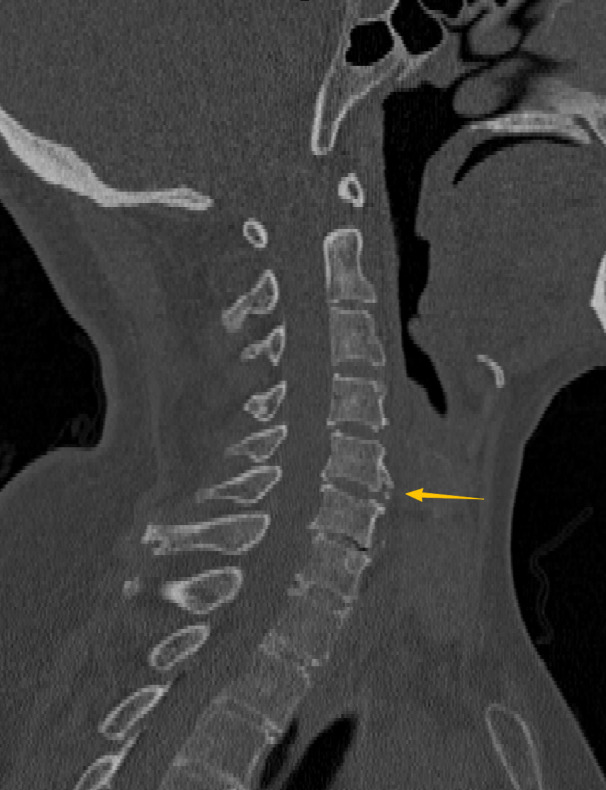
Preoperative sagittal cervical computed tomography. Sagittal CT image of the cervical spine demonstrates multilevel disc-space narrowing and endplate osteophyte formation across C4–7. The yellow arrow highlights representative ventral degenerative bony change contributing to canal compromise.

Baseline laboratory evaluation was largely unremarkable on routine testing. Urinalysis showed ketone bodies (2+) with abnormal urine coloration noted on the report. After the intraoperative finding of black-pigmented disc material, directed clinical re-evaluation revealed blue–gray pigmentation near the scleral limbus and auricular cartilage. In addition, the urine specimen showed marked darkening after standing, a finding clinically compatible with ochronuria (**Figures 3**–**5**). Given the clinical–radiographic progression and objective myelopathic signs, operative decompression was recommended.

**Figure 3:**
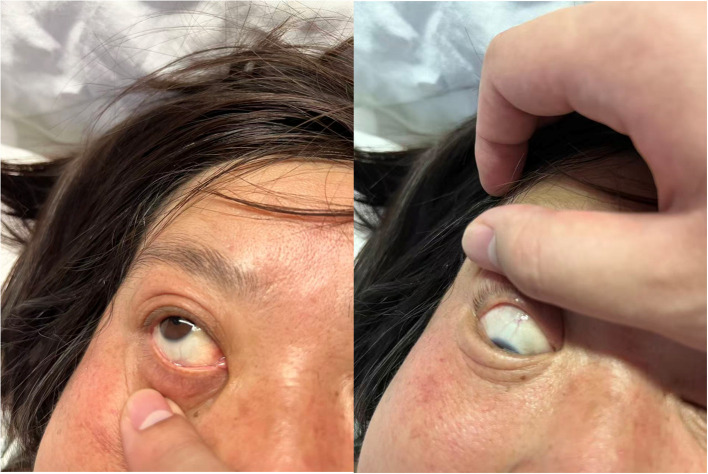
Ocular findings suggestive of ochronosis. Clinical photographs of the eyes show blue–gray pigmentation near the scleral limbus, a finding compatible with ocular ochronosis in the setting of clinically suspected alkaptonuria.

**Figure 4:**
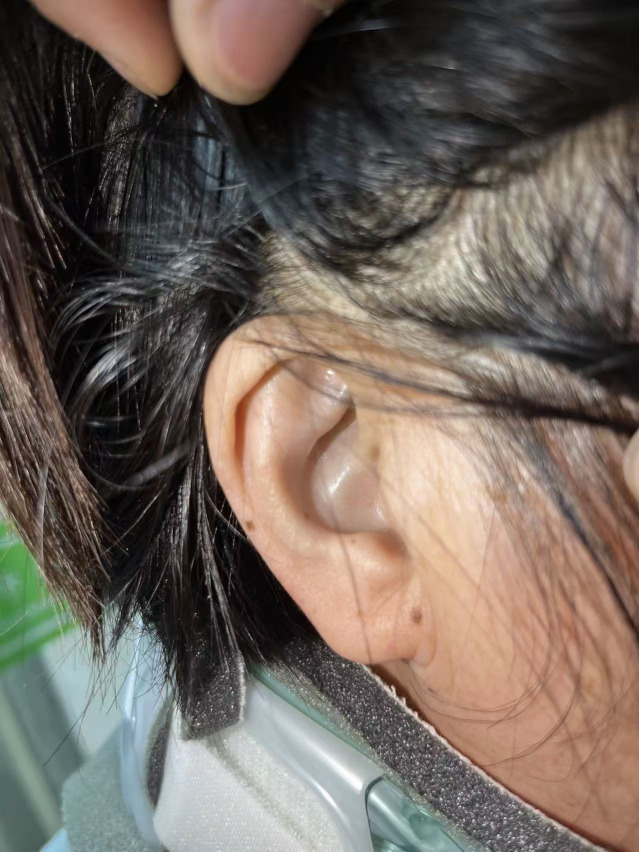
Auricular pigmentation. Clinical photograph shows bluish–gray pigmentation of the auricular cartilage/helix, supporting systemic connective-tissue pigmentation compatible with ochronosis.

**Figure 5:**
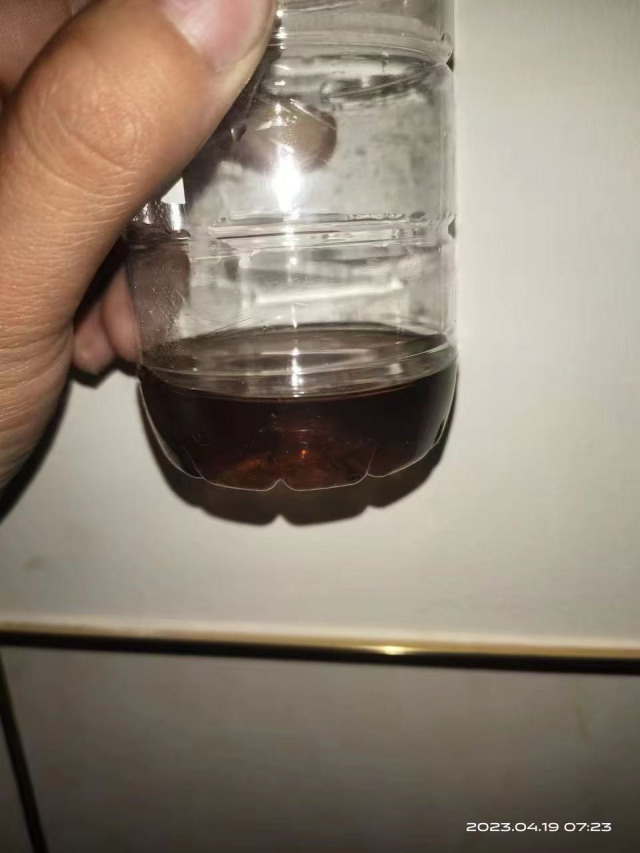
Urine darkening after standing. Photograph of the urine specimen demonstrates marked dark brown discoloration after standing, compatible with ochronuria in the setting of clinically suspected alkaptonuria. Definitive diagnosis requires quantitative urinary homogentisic acid measurement and/or HGD genetic testing.

### Therapeutic intervention

The patient underwent three-level anterior cervical discectomy and fusion (ACDF) from C4 to C7, including C4/5, C5/6, and C6/7 discectomy, ventral decompression, interbody cage placement, and anterior plate–screw fixation. Intraoperatively, the operative field revealed a striking and unexpected finding: the intervertebral discs and excised nucleus pulposus were diffusely black-pigmented, a macroscopic appearance strongly suggestive of ochronotic disc involvement (**Figures 6**, **7**). Representative nucleus pulposus specimens were collected (including for microbiological testing); bacterial and fungal cultures showed no growth. Histopathological examination and quantitative urinary HGA testing were recommended after surgery; however, these confirmatory tests were not available before manuscript preparation.

**Figure 6:**
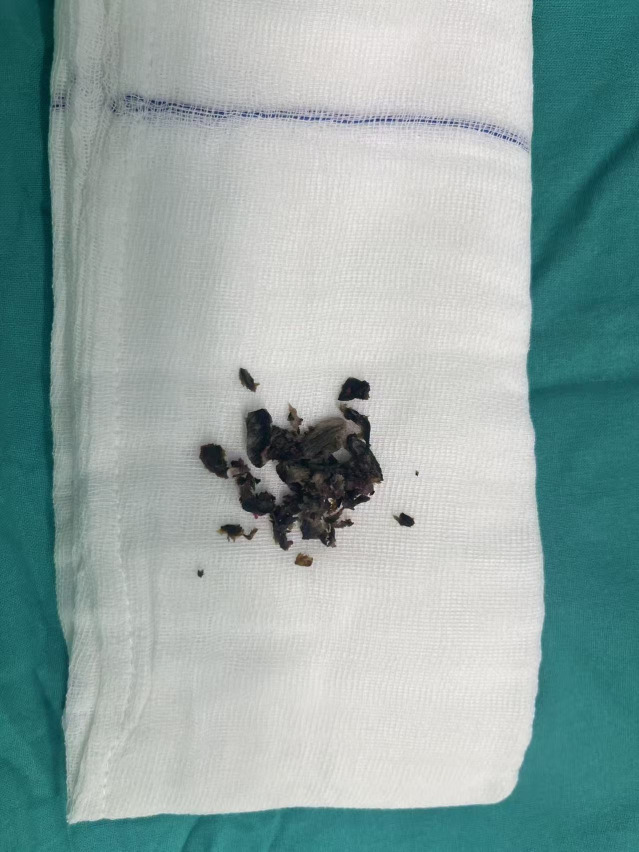
Gross appearance of the excised nucleus pulposus. Gross specimen photograph shows multiple excised nucleus pulposus fragments with diffuse black discoloration. In the appropriate clinical context, this finding is compatible with ochronotic disc pigmentation and should prompt evaluation for alkaptonuria-associated ochronosis.

**Figure 7:**
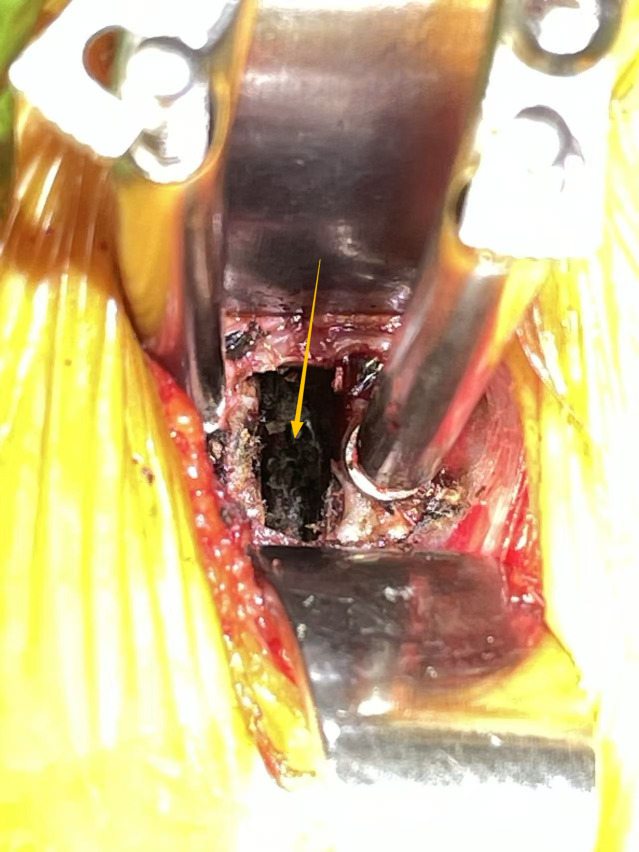
Intraoperative finding during anterior cervical discectomy. The intraoperative photograph demonstrates black-pigmented disc/nucleus material within the cervical disc space. The yellow arrow indicates the pigmented disc material, which represented the key macroscopic clue leading to suspicion of ochronotic involvement.

### Follow-up and outcomes

Postoperatively, the patient recovered uneventfully, without dysphagia, wound infection, neurological deterioration, or implant-related complications. At early postoperative assessment, limb numbness improved compared with the preoperative status, and gait stability was subjectively better than before surgery. Postoperative lateral cervical radiography confirmed satisfactory interbody cage position, anterior plate–screw fixation, and segmental alignment after C4–7 ACDF (**Figure 8**). She was discharged in stable condition with a cervical protection/rehabilitation plan and scheduled follow-up.

**Figure 8:**
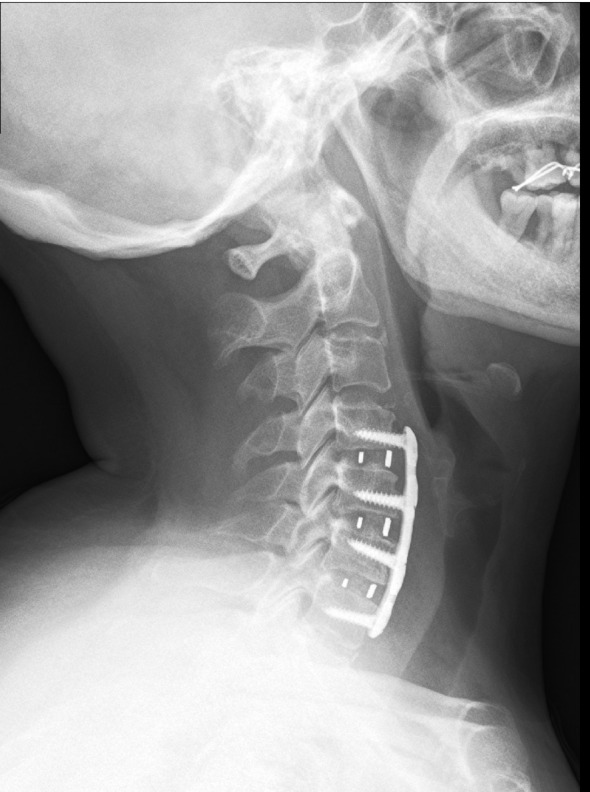
Postoperative lateral cervical radiograph after three-level C4–7 anterior cervical discectomy and fusion. The lateral radiograph demonstrates interbody cages and anterior plate–screw fixation spanning C4–7, with satisfactory implant position, segmental alignment, and restoration of anterior column support.

### Patient perspective

The patient reported that the progressive numbness of both upper limbs and the cotton-like sensation during walking had caused substantial anxiety before surgery. After the operation, she felt that limb numbness and walking stability had improved, and she expressed relief after being informed that the unusual black discoloration of the disc material might indicate an underlying metabolic disorder requiring further evaluation. She agreed to publication of the clinical details and anonymized images to increase awareness of this rare condition among clinicians.

## Discussion

The present case illustrates an uncommon but clinically important pathway to cervical myeloradiculopathy: multilevel cervical disc degeneration and herniation in a patient with clinical features highly suggestive of AKU-associated ochronosis, culminating in three-level C4–7 ACDF. The key intraoperative clue was the striking black discoloration of the intervertebral disc and nucleus pulposus, a macroscopic finding that should immediately raise suspicion for ochronosis, even when AKU has not been previously diagnosed. Importantly, this macroscopic appearance should trigger a diagnostic workup rather than be treated as definitive evidence, because biochemical confirmation with quantitative urinary HGA measurement remains necessary whenever feasible. Similar “black disc” observations have been repeatedly emphasized as the moment when an unrecognized metabolic disease becomes apparent to the spine surgeon (3).

### Mechanistic interpretation: why AKU can drive disc failure and neurologic compromise

AKU is caused by deficiency of homogentisate 1,2-dioxygenase, leading to lifelong accumulation of homogentisic acid (HGA). With oxidation, HGA forms reactive quinone intermediates (often described in the literature as benzoquinone-acetic-acid–related pigment) that polymerize into melanin-like ochronotic pigment and bind avidly to collagen-rich tissues. This process underlies the characteristic melanuria (urine darkening after standing/alkalinization) and progressive connective tissue discoloration (ochronosis).

From a spine perspective, ochronotic pigment deposition within the disc (annulus and nucleus) is not merely cosmetic. Multiple reports describe pigment-associated cross-linking, loss of elastic recoil, tissue brittleness, chronic inflammation/foreign-body reaction, and a propensity toward dystrophic mineralization—changes that plausibly convert the disc into a mechanically fragile structure prone to fissuring, extrusion, and segmental collapse. Clinically, AKU spinal disease most often manifests as multilevel spondyloarthropathy with disc space narrowing, calcification, vacuum phenomena, and stenosis; cervical involvement is less frequently reported but is increasingly recognized, including cases requiring decompression and fusion.

The “asymmetry” sometimes observed in AKU-related degeneration can be interpreted through a combined biochemical–biomechanical lens: heterogeneous pigment burden across segments and within disc compartments, superimposed on local loading patterns, may create focal stress risers that precipitate segment-dominant herniation (e.g., a large extrusion at one level) against a background of generalized multilevel disc degeneration.

### Comparison with prior literature

To reduce selection bias in the narrative literature review, we performed a targeted search of PubMed, Embase, Scopus, Web of Science, and Google Scholar from database inception to May 2026. The following search terms were used alone and in combination: “alkaptonuria,” “ochronosis,” “black disc,” “disc herniation,” “cervical disc,” “cervical myelopathy,” “spine,” “ACDF,” and “homogentisic acid.” Eligible articles included human case reports, case series, and literature reviews describing AKU/ochronosis with spinal disc involvement, especially cases with intraoperative black-pigmented disc material or surgically treated neurological compression. Articles without spinal involvement, non-human studies, non-AKU exogenous ochronosis, duplicate reports, and articles lacking sufficient clinical or operative detail were excluded. Reference lists of relevant reports and reviews were also screened manually. Ramirez-Velandia et al. recently reviewed 25 reported patients with AKU/ochronosis and spinal disc involvement published between 1963 and 2022, confirming that most surgically reported cases involved the lumbar spine, whereas cervical disc herniation with myelopathy remains distinctly uncommon (8).

Previously reported cervical cases suggest two recurring patterns. First, the black-disc finding may be the initial diagnostic trigger in patients without a known history of AKU, as illustrated by Broekx et al., who described black nucleus pulposus during C6–7 anterior surgery in an otherwise phenotypically unremarkable patient. Second, in patients with known or clinically apparent AKU, cervical disc extrusion can produce acute or progressive cord compression requiring anterior decompression, as reported by Sampaio Júnior et al. Compared with these cases, the present patient is notable for multilevel C4–7 anterior compression requiring three-level ACDF, together with ocular, auricular, and urinary findings clinically compatible with systemic ochronosis. However, unlike reports with quantitative HGA confirmation, the etiologic attribution in our case remains clinically probable if biochemical testing is unavailable (**Table 1**).

**Table 1:** Selected published surgical reports of “black disc”/spinal disc pigmentation in AKU (representative cases).

Study (year)	Region/level	Presentation	Procedure	Key intraoperative clue	Diagnostic confirmation/outcome
Broekx et al. (2024)	Cervical (C6–7)	Critical canal stenosis; surgery performed to prevent myelopathy	C6–7 anterior microdiscectomy + interbody fusion	Black nucleus pulposus, otherwise no obvious phenotype	Course uneventful; authors emphasize black disc should prompt AKU workup
Sampaio Júnior et al. (2025)	Cervical (C5–6; DDD also at C4–5)	Acute severe neck pain with neurologic signs (incl. Hoffmann); large extruded disc with cord compression	C5–6 discectomy + anterior arthrodesis	Ochronotic disc pigmentation	Urinary HGA markedly elevated; early strength improvement and discharge day 5
Ösün et al. (2017)	Lumbar (L4–5)	Radiculopathy with weakness; extruded herniation	L4–5 microdiscectomy	Degenerated black disc	Retrospective history (dark urine, auricular pigmentation) + urine HGA positive; no postoperative deficit

### Diagnostic pitfalls and differential diagnosis

A black or darkly pigmented disc can be misattributed to nonspecific degeneration, old hemorrhage, or iatrogenic staining. Two pitfalls deserve emphasis:

1. Assuming routine urinalysis is diagnostic. Dipstick findings are nonspecific; AKU requires definitive quantification of urinary HGA (commonly by chromatographic mass spectrometry methods) and/or molecular confirmation (9).
2. Overreliance on “classic phenotype.” Some patients undergoing cervical surgery may lack obvious cutaneous/auricular/scleral findings at the time of presentation. In Broekx et al., no macroscopic abnormalities of skin, muscles, or ligaments were noted, yet the nucleus pulposus was black (3).

The broader differential for connective tissue darkening includes exogenous ochronosis (e.g., certain phenolic compounds), which is discussed in the ochronosis literature, reinforcing the need for biochemical confirmation rather than visual impression alone.

### Management rationale and perioperative considerations

The indication for surgery in this case followed conventional cervical myelopathy/myeloradiculopathy principles: progressive neurologic dysfunction with imaging-confirmed multilevel ventral compression and cord signal change. Multilevel ACDF (C4–7) is a standard strategy when pathology is predominantly anterior (disc–osteophyte complex, focal disc protrusions) and when restoration of disc height and alignment is desirable (10–12).

Key intraoperative decision points in suspected AKU:

Recognize and document the black disc/nucleus pulposus (photography is valuable for publication and teaching).

Send disc material for histopathology, which may show pigment deposition with degenerative changes; this can support the diagnosis when metabolic testing is delayed.

Trigger a directed systemic evaluation: a focused history for urine darkening after standing, renal stones, tendon/ear pigmentation, and family history; and referral for confirmatory metabolic/genetic testing.

Definitive metabolic testing recommended after intraoperative suspicion:

Quantitative urinary HGA measurement (diagnostic cornerstone).

Consider genetic testing for pathogenic variants in the HGD gene when available and for family counseling.

Perioperatively, surgeons should remember that AKU is systemic. Reviews and case series emphasize associations with renal stones and connective tissue involvement; therefore, anesthesia and perioperative teams should consider targeted screening where clinically indicated (e.g., renal/urogenital history, cardiac evaluation if symptoms/history suggest valvular involvement).

### Limitations

This report has several limitations. First, if quantitative urinary HGA measurement and HGD genetic testing are not available, the diagnosis of AKU-associated ochronosis should be regarded as clinically probable rather than biochemically confirmed, despite the highly suggestive combination of black-pigmented disc material, ocular/auricular pigmentation, and urine darkening after standing. Second, the follow-up period was limited to early postoperative assessment, and standardized longitudinal outcome measures such as JOA, mJOA, NDI, or VAS scores were not systematically obtained. Third, histopathological confirmation of ochronotic pigment deposition in the excised disc material was not performed, which further limits the strength of etiologic attribution. Finally, as a single-patient report, this case cannot define the optimal surgical approach or long-term prognosis for the heterogeneous population of patients with AKU-related spinal disease.

### Take-home messages (learning points)

A black intervertebral disc/nucleus pulposus is a red-flag intraoperative sign that should prompt evaluation for alkaptonuria/ochronosis, even when classic pigmentation is absent.

AKU likely promotes disc degeneration via HGA oxidation → pigment deposition → collagen cross-linking/brittleness and degeneration, predisposing to herniation and stenosis.

Definitive diagnosis requires quantitative urinary HGA and/or genetic confirmation—routine urinalysis alone is insufficient.

Standard cervical decompression principles apply; reported surgical outcomes are generally satisfactory when the compressive pathology is addressed appropriately.

## Conclusion

This case highlights an uncommon cervical presentation clinically suggestive of AKU-associated ochronosis, in which multilevel cervical disc degeneration culminated in symptomatic cervical myeloradiculopathy requiring three-level C4–7 ACDF. The intraoperative finding of a diffusely black intervertebral disc and nucleus pulposus is a highly characteristic and clinically actionable clue; when encountered unexpectedly during routine cervical decompression, it should prompt the surgical team to consider ochronosis and initiate a directed diagnostic pathway. When confirmatory metabolic testing is unavailable, the case should be interpreted primarily as a diagnostic alert emphasizing recognition, documentation, tissue sampling, and referral for definitive metabolic evaluation.

For spine surgeons, the practical implication is twofold: first, standard principles of decompression and stabilization remain effective for neurological compromise; second, surgery addresses the compressive phenotype but does not replace definitive metabolic evaluation. Definitive confirmation should rely on quantitative urinary homogentisic acid testing (with genetic confirmation where available), followed by multidisciplinary assessment for systemic involvement and long-term surveillance for adjacent-segment degeneration and other ochronosis-related complications.

## Data Availability

The original contributions presented in the study are included in the article/supplementary material. Further inquiries can be directed to the corresponding authors.
